# How do you perceive threat? It’s all in your pattern of brain activity

**DOI:** 10.1007/s11682-019-00177-6

**Published:** 2019-08-24

**Authors:** Orlando Fernandes, Liana Catrina Lima Portugal, Rita de Cássia S. Alves, Tiago Arruda-Sanchez, Eliane Volchan, Mirtes Garcia Pereira, Janaina Mourão-Miranda, Letícia Oliveira

**Affiliations:** 1 Laboratory of Neuroimaging and Psychophysiology, Department of Radiology, Faculty of Medicine, Federal University of Rio de Janeiro, 255 Rodolpho Paulo Rocco st., Ilha do Fundão, Rio de Janeiro, RJ 21941-590 Brazil; 2 Laboratory of Behavioral Neurophysiology, Department of Physiology and Pharmacology, Biomedical Institute, Federal Fluminense University, Niterói, RJ Brazil; 3 IBMR University Center, Rio de Janeiro, RJ Brazil; 4 Laboratory of Neurobiology II, Institute of Biophysics Carlos Chagas Filho, Federal University of Rio de Janeiro, Rio de Janeiro, RJ Brazil; 5 Centre for Medical Image Computing, Department of Computer Science, University College London, London, UK; 6 Max Planck University College London Centre for Computational Psychiatry and Ageing Research, University College London, London, UK

**Keywords:** MVPA, fMRI, Decoding, Threat, Perception

## Abstract

Whether subtle differences in the emotional context during threat perception can be detected by multi-voxel pattern analysis (MVPA) remains a topic of debate. To investigate this question, we compared the ability of pattern recognition analysis to discriminate between patterns of brain activity to a threatening versus a physically paired neutral stimulus in two different emotional contexts (the stimulus being directed towards or away from the viewer). The directionality of the stimuli is known to be an important factor in activating different defensive responses. Using multiple kernel learning (MKL) classification models, we accurately discriminated patterns of brain activation to threat versus neutral stimuli in the directed towards context but not during the directed away context. Furthermore, we investigated whether it was possible to decode an individual’s subjective threat perception from patterns of whole-brain activity to threatening stimuli in the different emotional contexts using MKL regression models. Interestingly, we were able to accurately predict the subjective threat perception index from the pattern of brain activation to threat only during the directed away context. These results show that subtle differences in the emotional context during threat perception can be detected by MVPA. In the directed towards context, the threat perception was more intense, potentially producing more homogeneous patterns of brain activation across individuals. In the directed away context, the threat perception was relatively less intense and more variable across individuals, enabling the regression model to successfully capture the individual differences and predict the subjective threat perception.

**Electronic supplementary material:**

The online version of this article (10.1007/s11682-019-00177-6) contains supplementary material, which is available to authorized users.

## Introduction

Functional neuroimaging (fMRI) has provided a great opportunity to obtain insight into of how emotional representations are encoded in brain activity (Lindquist et al. 2012; Murphy et al. 2003). The most common approach used to analyze fMRI data is based on the general linear model (GLM) (Friston et al. 1995) and is known as a mass-univariate analysis because it makes statistical inferences independently at each location (voxel). Despite the success of this approach in identifying brain regions associated with a specific stimulus or cognitive task, mass-univariate analysis does not take into account the multivariate aspect of data, i.e., the fact that each scan contains information about brain activation at thousands of voxels that are related to each other. In contrast, multivariate approaches simultaneously consider information across multiple voxels in the brain and, therefore, can provide higher sensitivity for identifying subtle differences in brain activation. Consequently, multivariate analyses can improve our ability to obtain valuable information from the whole brain based on functional neuroimaging.

In the last fifteen years, pattern recognition, specifically multi-voxel pattern analysis (MVPA), has been increasingly used as an alternative approach for analyzing neuroimaging data (Cox and Savoy 2003; Haxby et al. 2001; Haynes and Rees 2005; LaConte et al. 2005; Mourão-Miranda et al. 2005). Pattern recognition analyses use computer-based techniques to automatically discover regularities in the data, i.e., patterns (Shawe-Taylor and Cristianini 2004). These discovered pattern regularities in existing datasets can then be used to make predictions for new datasets, such as to classify an independent series of individuals, case by case, into different categories (patient x control) or different brain states (emotional x neutral). Pattern recognition techniques can also be used to predict continuous measures such as behavioral scores or symptom levels (Portugal et al. 2016).

One remarkable disadvantage of MVPA is the difficulty in making local inferences because the model is based on the entire multivariate pattern or set of voxels included in the model. Therefore, it is not possible to make inferences at the voxel level, making difficult to discuss the relative importance of individual brain regions in whole-brain MVPA models. This issue can be addressed using a multiple kernel learning (MKL) model that considers the whole-brain multivariate pattern as a combination of regional patterns and learns the contribution of each brain region for the predictive task (i.e., classification or regression, Schrouff et al. 2018). Regions that carry more information about the variable being predicted will have a higher contribution to the model, which is characterized by the kernel or region weights. Brain regions can then be ranked according to their contribution to the model, which facilitates the interpretation of the predictive model in terms of the contributions of different brain regions.

Previous studies have demonstrated that distributed patterns of brain activity measured with fMRI contain information capable of differentiating among emotional brain states (Baucom et al. 2012; Chang et al. 2015; Johnson et al. 2015; Kragel et al. 2016; Kragel and LaBar 2015; Markey et al. 2013; Saarimäki et al. 2016; Yuen et al. 2012). For instance, Kassam et al. (2013) classified nine different emotions based on patterns of brain activity during self-induced emotional states with an 84% mean rank accuracy (the normalized rank of the correct label, averaged for each fold) when training and testing were performed with the same subject and with a 70% mean rank accuracy when training and testing were performed with independent subjects (considering 50% chance level). In this same line, Saarimäki et al. (2016) showed that MVPA could discriminate patterns of brain activity among six discrete emotions (disgust, fear, happiness, sadness, anger, and surprise) induced by short movies or mental imagery. Moreover, MVPA has also been applied to predict continuous measures in emotional contexts (Chang et al. 2015; Fernandes et al. 2017). Chang et al. (2015) identified a neural signature that predicted differences in the subjective experience of negative emotion from patterns of fMRI activity and recent work from our group demonstrated that pattern regression analyses can be used to decode a personality trait from patterns of brain activation during emotional stimuli (Fernandes et al. 2017).

In summary, the high sensitivity of MVPA approaches may facilitate significant progress toward discovering links between distributed brain representations and discrete or continuous emotions. Nevertheless, limited work has investigated the roles of different emotional contexts in the discriminability among brain states using MVPA. For example, does the accuracy of MVPA for discriminating emotional and neutral brain states vary according to the threat level induced by different threat contexts? To investigate this question, we compared the ability of MVPA to discriminate between brain activity patterns to threatening stimuli (a person holding a gun) versus brain activity patterns to neutral stimuli (a person holding a non-lethal object) in two different contexts. The two contexts differed only in the direction of the stimuli, i.e., the threating and neutral objects were either directed towards the viewer (*directed towards context*) or directed away from the viewer (*directed away context*). The direction of the threat is a subtle change in the visual scene that may increase the perception of threat imminence, i.e., whether the threat is directed towards or away from the viewer. Many studies in the literature have shown that the direction of the threat can modulate psychophysiological responses and brain activity (Dimberg and Ohman 1983; Flykt et al. 2007; Hugdahl and Johnsen 1989; Kveraga et al. 2015). For example, Flykt et al. (2007) tested the effect of threat direction in conditioning experiments using conditioned biological threats (e.g., snakes) or cultural threats (e.g., guns). The results revealed that threatening stimuli directed towards the viewer produced conditioned skin conductance responses that were resistant to backward masking, regardless of whether the threat was due to biological or cultural causal factors. Threat stimuli directed away from the viewer produced conditioned skin conductance responses, but backward masking abolished this effect. The authors emphasized that the direction of the threat stimulus was the critical factor modulating defensive responses and that threat stimuli directed towards the viewer increased threat imminence, which enhanced psychophysiological responses. In a very interesting paper, Grèzes et al. (2013) found that “self-directed body expressions” of anger (those directed towards the viewer) triggered higher corrugator reactivity and greater feelings of threat than “other-directed bodies” (directed away from the viewer). In addition, the direction of the threat stimulus has been considered a key factor in activating different defensive responses (Bastos et al. 2016; Carlson et al. 2009; Dimberg and Ohman 1983; Fernandes et al. 2013; Flykt et al. 2007; Hugdahl and Johnsen 1989). Pictures of a person pointing a gun towards the viewer were reported as significantly more threatening than those of a person pointing a gun away from the viewer, the former eliciting more intense magnitudes of threat, proximity, situation inescapability, and impossibility of hiding (Bastos et al. 2016; Fernandes et al. 2013). Moreover, threat directed-towards pictures were reported as significantly less ambiguous than threat directed-away pictures (Bastos et al. 2016).

In a previous behavioral study from our group (Fernandes et al. 2013), we demonstrated that directed-towards pictures produced a decrease in reaction time compared to neutral stimuli, while the opposite pattern was observed for threat directed-away pictures, i.e., an increase in reaction time compared to neutral pictures. This reduction in response time when stimuli were directed towards the viewer was attributed to increased motor preparation resulting from strong activation of the defense response cascade. Threat directed-away stimuli possibly activated the defense cascade, although with less intensity, prompting immobility, leading to an increase in response time (Fernandes et al. 2013). It is reasonable to hypothesize that individual threat perception modulates the patterns of brain reactivity in each context. Thus, a critical question is whether the individual’s subjective threat perception rating can be decoded from their patterns of brain activity. Considering the importance of threat direction in modulating defensive responses, we hypothesized that a higher accuracy would be achieved for discriminating between emotional versus neutral brain states in the directed towards context than in the directed away context. Moreover, we also hypothesized that the individual threat perception could be decoded from the pattern of brain activation to threatening stimuli in both contexts. To investigate these hypotheses, we applied an MKL classification model to discriminate between patterns of brain activation to threat versus neutral stimuli in two different contexts (*directed towards* and *directed away*) and an MKL regression model to decode the individual’s subjective threat perception from his/her brain activation pattern to threat in both contexts.

## Methods

### Participants

Thirty-eight undergraduate or graduate students without history of neurological or psychiatric illness and who were not taking any central nervous system-acting medication participated in this study (18 women; age range: 18–38 years, average = 26.9 (SD = 4.9) years). All participants had normal or corrected-to-normal vision, and each participant provided written informed consent prior to participation. Part of the fMRI dataset used in this study was previously described in Fernandes et al. (2017). The project was performed in accordance with the local Ethics Committee of the Federal Fluminense University, Brazil.

### Data acquisition

The data for this study were collected at the Department of Radiology at Hospital Universitário Clementino Fraga Filho (Federal University of Rio de Janeiro, Brazil) using a 1.5-T Siemens (Magnetom Avanto) scanner with a 8-channel head coil for brain imaging. The fMRI runs were acquired with a sequential ascending framework using a gradient-echo echo-planar imaging (EPI) single-shot sequence covering 25 axial slices (4 mm thick; 0.6 mm gap; TR/TE = 2000/40 ms; IST = 80 ms; FOV = 256 mm; matrix, 64 × 64; voxel dimensions, 4 × 4 × 4 mm). Head movements were restrained by foam padding. In each run, 198 functional volumes were acquired in four runs. In addition, a three-dimensional high-resolution T1-weighted anatomical image (TR/TE = 2730/3.27 ms, 128 slices, 0.6 mm gap, FOV = 250 mm, voxel dimensions 1.33 × 1 × 1.33 mm) was obtained at the beginning of each session for functional to anatomical image registration.

### Stimuli

Eighty-four pictures comprising two sets, threat and neutral stimuli (42 pictures each), were employed in this study. The threatening stimuli were photographs of a man holding a gun. The neutral stimuli were photographs of a man holding a non-lethal object, such as a camera or a domestic tool. The guns and the neutral objects were either directed towards or away from the viewer (21 pictures in each). In summary, the stimulus categories were as follows: (1) threat stimulus directed towards, (2) threat stimulus directed away, (3) neutral stimulus directed towards, and (4) neutral stimulus directed away. The pictures were matched in several properties to avoid confounding effects unrelated to emotion on brain activity (Steinmetz et al. 2011). The ethnicity of the men holding the objects was balanced among the categories. Threat and neutral stimuli were matched in terms of brightness, contrast, spatial frequency and complexity (Table 1). Bradley et al. (2007) studied event-related potentials in response to emotional pictures and showed that, beyond the affective content of pictures, the complexity of pictures also impacts the amplitude of brain potentials. We attempted to minimize this confounding factor by selecting only emotional and neutral stimuli with approximately the same level of complexity, i.e., they were all clear figure-ground pictures (in which the principal object/person was easily distinguishable from a uniform background). To assess the adequacy of this a priori selection, we followed the procedures of Bradley et al. (2007) and asked an independent sample of 58 students (42 female) to rate picture complexity on a scale of 1–9 (1 = clear figure-ground, 9 = complex scenes). In order to investigate significant differences in the physical characteristics of stimuli, we performed a repeated measure ANOVA with two factors: direction of stimuli (towards x away) and category (threat x neutral) to each individual physical characteristic. Only for *contrast*, ANOVA revealed a significant main effect of direction (F (1,20) = 11.662, *p* = 0.003), showing that the directed towards stimuli (threat and neutral) presented more contrast than directed away stimuli (threat and neutral). As we used pattern classification analysis to investigate whether it was possible to discriminate between patterns of brain activity to threat versus neutral stimuli *within* each context, this difference is not very critical. No other main effects were observed due to brightness, spatial frequency and complexity.

**Table 1:** **Stimuli evaluation report:** Threat and neutral stimulus ratings for valence, arousal, complexity, and physical characteristics of the pictures (brightness, contrast, and spatial frequency) in the directed towards and directed away contexts. The threat perception index to threat stimuli are presented for the directed towards and directed away contexts

	Directed towards context		Directed away context	
	Threat	Neutral	Threat	Neutral
*Threat perception index*	12.38 (7.47)	–	10.29 (7.94)	–
*Valence*	2.06 (1.22)	5.20 (0.99)	2.73 (1.37)	5.47 (1.01)
*Arousal*	6.56 (2.20)	3.65 (2.02)	5.50 (2.16)	3.63 (1.99)
*Complexity*	3.00 (0.74)	2.92 (0.57)	2.59 (0.77)	3.02 (1.04)
*Brightness*	76.47 (23.75)	79.33 (24.98)	92.84 (36.86)	87.07 (37.10)
*Contrast*	25.43 (9.73)	27.67 (8.78)	20.95 (10.57)	21.42 (7.37)
*Spatial frequency*	0.96 (0.10)	0.99 (0.14)	0.99 (0.06)	1.02 (0.11)

Standard deviations are shown within parentheses. Brightness, contrast and spatial frequency were measured according to Bradley and colleagues (Bradley et al. 2007). Brightness was defined as the mean RGB (red, green and blue) value for each pixel, averaged across all pixels for each picture. Contrast was defined as the standard deviation of the mean RGB values computed across pixels for each column. Spatial frequency was defined as the median fast Fourier transform (FFT) power, which was computed for each row and column and then averaged. The valence, arousal, complexity ratings, brightness, contrast and spatial frequency values have been already published for the threat and neutral stimulus in a previous study of our group (Fernandes et al. 2017).

The pictures were obtained from the World Wide Web, purchased from Getty Images® (http://www.gettyimages.com), selected from the International Affective Pictures System (IAPS, Lang et al. 2005) and produced by the authors with the aid of a professional photographer. In order to minimize confounding effects related to facial features, such as gaze direction, we used a blur effect in imaging processing software (Adobe Photoshop©) to blur the faces. All pictures had the same size (1024 × 768 pixels). Following the protocol developed by Lang and colleagues (Lang et al. 1997), the pictures were rated on a scale of 1–9 in terms of pleasure and arousal by a separate group of 134 participants (104 female, 21.5 years ±3.36) using the paper-and-pencil version of the Self-Assessment Manikin (Bradley and Lang 1994). The means and standard deviation values of valence and arousal for each category are shown in Table 1. To assess whether the ratings for valence and arousal differ between the categories, we performed a repeated measures ANOVA with two factors: direction of stimuli (towards x away) and category (threat x neutral) separately for valence and arousal ratings. For *valence*, ANOVA revealed a significant main effect of direction (F(1,20) = 18.561, *p* < 0.001) and a main effect of valence (F(1,20) = 400.162, p < 0.001). As expected, the threat stimuli were considered more negative than neutral stimuli for both directed towards and directed away contexts. Furthermore, the directed towards stimuli (threat and neutral) were considered more negative than directed away stimuli (threat and neutral). No significant interaction was found. For *arousal*, ANOVA revealed a significant main effect of direction (F(1,20) = 8.573, *p* = 0.008) and a main effect of valence (F(1,20) = 147.482, p < 0.001) in the same line of the valence results. However, in this case, we found a significant interaction between the two factors (F(1,20) = 8.151, *p* = 0.009). The post hoc Newman-Keuls tests revealed that threat stimuli directed towards were more arousing than the threat stimuli directed away (p < 0.001), no difference was observed between directed towards and directed away neutral stimuli (*p* = 0.96).

### Experimental design

**Fig. 1:**
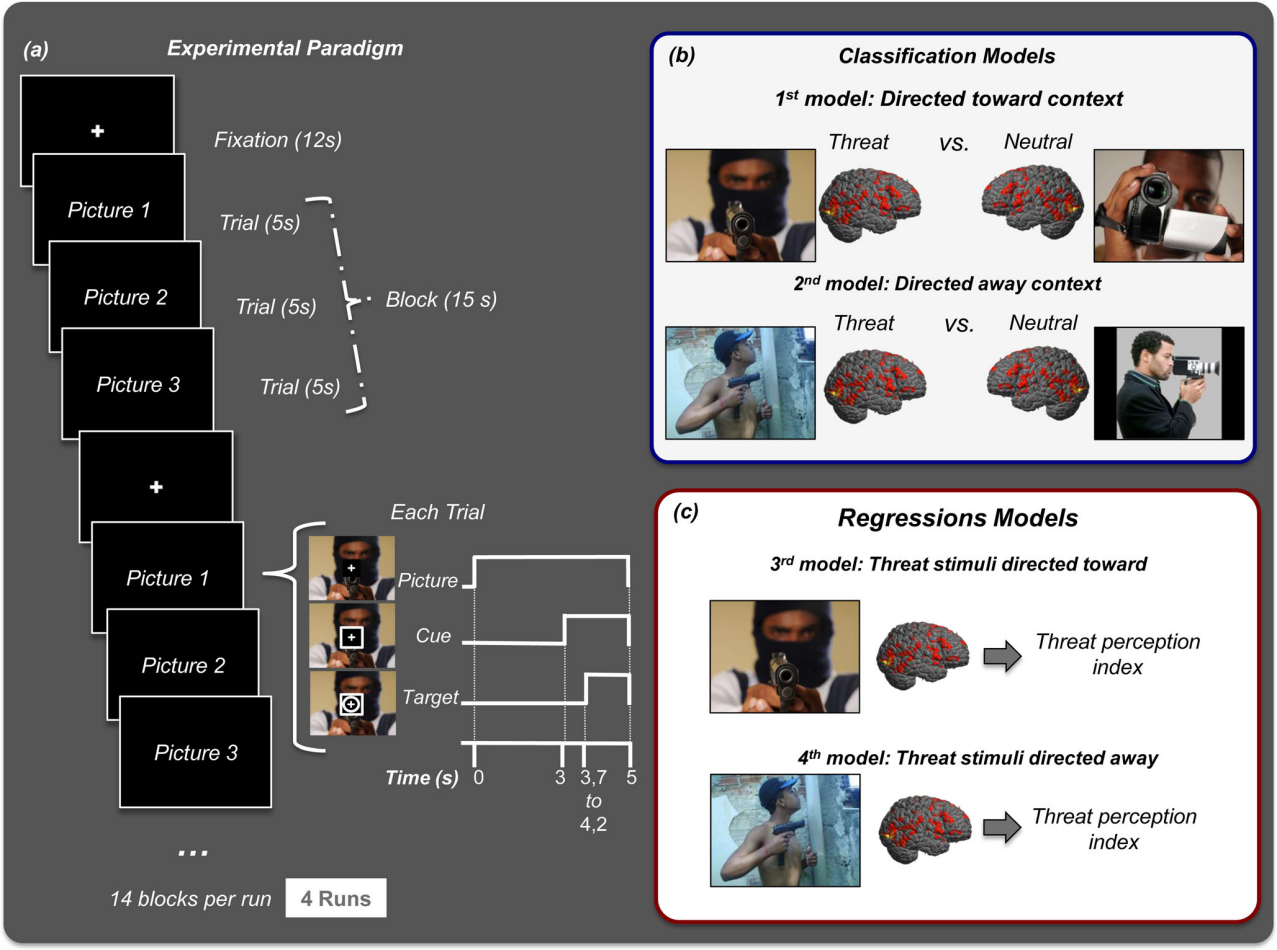
Experimental Design: (**a**) Experimental paradigm – Each trial began with the presentation of a photograph and a fixation spot. After 3 s, a square appeared around the fixation spot 700–1200 ms prior to target onset. The target was a small annulus that appeared around the fixation spot. The total duration of the trial was 5 s. Each block consisted of three photographs of the same category. The fixation cross on a black background remained for 12 s. The experimental session consisted of 56 blocks (14 blocks for each category), pseudo randomized through the experiment and divided into 4 runs. (**b**) MKL classification models - The first model was trained to discriminate between the patterns of brain activity to threat versus neutral stimuli in the directed towards context. The second model was trained to discriminate between the patterns of brain activity to threat versus neutral in the directed away context. (**c**) MKL regression models - Two regression models were trained with the goal of predicting the subjects’ threat perception indices. The first model was based on the patterns of brain activation to threat stimuli directed towards the viewer, and the second model was based on patterns of brain activation to threat stimuli directed away from the viewer

The stimuli were projected onto a screen located in front of the participant’s body and were viewed inside the scanner using a mirror attached to the head coil. Stimuli were presented using Presentation software (Neurobehavioral Systems, version 11.0, Inc., Albany, CA, USA). At the beginning of each trial, the participants were instructed to attend to each picture while maintaining their eyes fixed on a fixation spot at the center of the screen. After attending to the picture for 3 s, a square appeared around the fixation spot 700–1200 ms prior to the target onset. The target was a small annulus that appeared around the fixation spot. The picture, the square and the target remained visible until the end of the trial, which had a total duration of 5 s. The participants were required to press a button with their right index finger as quickly as possible after target onset. An MR-compatible response key, positioned on the right side of the participant’s abdomen, recorded the responses. Each block consisted of three pictures (5 s each) of the same category (threat stimulus directed towards or directed away and neutral stimulus directed towards or directed away) presented in sequence, followed by 12 s of presentation of the fixation cross. The experimental session consisted of 56 blocks (14 blocks of each category), pseudo randomized throughout the experiment, divided into 4 runs; Fig. 1 shows the experimental paradigm. Each picture was presented and repeated once, but not in the same run.

### Threat evaluation reports

After MRI acquisition, thirty-four subjects (16 women; average age = 26.3; SD = 4.6) evaluated the emotional impact of the stimuli from each set (i.e. threat and neutral). Four subjects were excluded from the pattern regression analysis because they did not complete the threat evaluation questionnaire. The threat evaluation questionnaire was answered using a paper and pencil on a Likert-like scale of 9 points. Questions were related to the dimensions of the threat perception: (i) threat magnitude, (ii) the distance between the threat and the participant (proximity), (iii) the escapability of the situation (inescapability) and (iv) the presence of available hiding places (impossibility of hiding). Participants were instructed to rate the stimuli from each set according to their subjective feelings when viewing the threat stimulus. These subjective threat dimensions were adapted from the studies of Blanchard et al. (2001) and Shuhama et al. (2008), together with others studies that have demonstrated that particular features of threatening stimuli that are determinant for triggering a defensive response in animals are very similar to defensive strategies in humans (Bastos et al. 2016; Fernandes et al. 2013; Perkins and Corr 2006). The ratings obtained for the four dimensions were summed and used as an index for threat perception; these values are presented in Table 1. The threat perception index related to each set of threatening stimuli (*directed towards* and *directed away*) were used as targets in the MKL regression models.

### Data pre-processing and general linear model analysis

The Statistical Parametric Mapping software package (SPM8, Wellcome Department of Cognitive Neurology, London, UK) was used for pre-processing and General Linear Model (GLM) analysis. The first three functional volumes of each run were removed to eliminate non-equilibrium effects of magnetization. The remaining images were corrected for head movement by realigning all the images to the first image via rigid body transformations. The data were realigned to remove residual motion effects. For each participant, functional and structural images were co-registered. Structural data were segmented and normalized by matching them to the standardized MNI template (Montreal Neurologic Institute, Evans et al. 1993), and the transformation parameters estimated in this step were applied to all functional images. Finally, the functional images were spatially smoothed with an 8-mm full-width half-maximum (FWHM) Gaussian filter.

GLM analysis was performed according to the framework implemented in SPM8 (Friston et al. 1995). For each participant, a GLM model was built with the four experimental conditions (threat directed towards, threat directed away, neutral directed towards and neutral directed away) entered into the design matrix as separate regressors. Before estimation via multiple regression, regressors of interest were convolved with a canonical hemodynamic response function. As our aim was to investigate whether the MKL algorithm could decode different emotional contexts, the 15-s experimental blocks were used as regressors of interest for each condition, and the 12-s fixation cross between the blocks served as a baseline. Movement parameters from the realignment step were entered as covariates of no interest to control for participant’s movement. The low frequency components were modelled by a set of discrete cosine functions (128-s cut-off period). The four runs were first modelled independently, and at the end, contrast images for each condition were created based on all runs: (1) threat directed towards > baseline, (2) threat directed away > baseline, (3) neutral directed towards > baseline, (4) neutral directed away > baseline. These contrast images represent the patterns of brain activation during each experimental condition with respect to baseline and were used as input to the pattern classification and regression analyses. We used one contrast image per participant in each classification (in this case one contrast image per class) and regression model.

We used a custom-created mask to exclude voxels, common to all participants that had “NaN” (not a number) in the contrast images. This mask can improve the performance of pattern recognition analyses by decreasing the number of non-informative features/voxels in the model (Portugal et al. 2016).

### Pattern recognition analysis

**Fig. 2:**
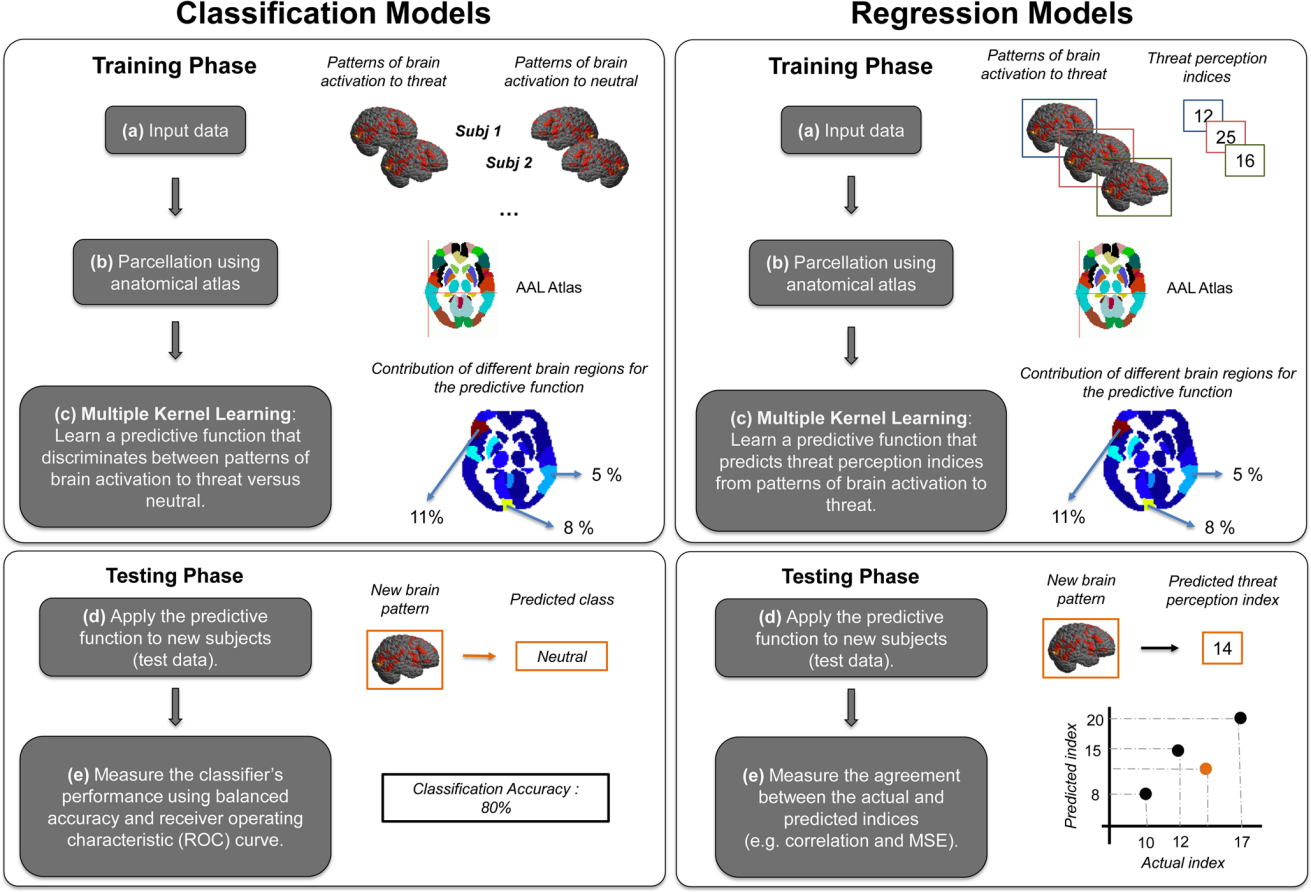
**Multiple Kernel Learning Framework. MKL Classification Model (left panel):** (**a**) The training data for the multiple kernel learning (MKL) classification model consists of examples that pair a contrast image from the GLM model and the corresponding label of the experimental conditions (threat or neutral). (**b**) The MKL framework uses a predefined anatomical template to segment the contrast images into 120 anatomical brain regions. (**c**) During the training the MKL model simultaneously learns the contribution of each region (kernel/regions weights) and within each region the contribution of each voxel (voxel weights) for the predictive function, respectively. (**d**) During the test phase, given the contrast image of a test subject the MKL model predicts its corresponding experimental condition (threat or neutral). (**e**) The classification model performance is evaluated using accuracy and ROC curve (see methods and results). **MKL Regression Model (right Panel):** (**a**) The training data for the multiple kernel learning (MKL) regression model consists of examples that pair a contrast image from the GLM model and the corresponding threat perception index. (**b**) The MKL framework uses a predefined anatomical template to segment the contrast images into 120 anatomical brain regions. (**c**) During the training the MKL model simultaneously learns the contribution of each region (kernel/regions weights) and within each region the contribution of each voxel (voxel weights) for the predictive function, respectively. (**d**) During the test phase, given the contrast image of a test subject the MKL model predicts its corresponding threat perception index. (**e**) The model performance is evaluated using three metrics that measure the agreement between the predicted and the actual threat perception indices: Pearson’s correlation coefficient (r), coefficient of determination (r^2^) and mean squared error (MSE)

Pattern recognition analyses were performed according to the framework implemented in the Pattern Recognition for Neuroimaging Toolbox (PRoNTo, Schrouff et al. 2013). We used pattern classification analysis to investigate whether it was possible to discriminate between patterns of brain activity to threat versus neutral stimuli in each context (*directed towards* and *directed away*). More specifically, we trained and tested two classification models. The first model was trained to discriminate between the patterns of brain activity to threat versus neutral stimuli in the directed towards context (‘*directed towards threat* versus *neutral’*). The second model was trained to discriminate between the patterns of brain activity to threat versus neutral in the directed away context (‘*directed away threat* versus *neutral*’). Additionally, we used pattern regression analysis to investigate the relationship between patterns of brain activity to threat in both contexts and the subjects’ threat perception indices. More specifically, we trained two regression models with the goal of predicting the subjects’ threat perception indices. One model was based on patterns of brain activation to threat directed towards the observer (‘*threat directed towards regression*’), and the other was based on patterns of brain activation to threat directed away from the observer (‘*threat directed away regression*’), see Fig. 2.

In the present work, we used a pattern recognition model that embeds prior information about brain anatomy. This approach relies on MKL (Schrouff et al. 2018) and models the whole-brain multivariate pattern as a combination of regional patterns based on a previously defined atlas. More specifically, the model learns the contribution of each brain region (kernel or regions weights) and the contribution of each voxel within each region (voxel weights) to build the prediction function. As the MKL model currently implemented in PRoNTo assumes sparsity in the kernel combination (Simple MKL, Rakotomamonjy et al. 2008), it will select a subset of the regions that are important for the predictive task (classification or regression), the remaining regions will have kernel weights equal to zero. We used the predefined automated anatomical labelling (AAL2) atlas of Tzourio-Mazoyer and colleagues (Tzourio-Mazoyer et al. 2002) that was recently updated with new parcellation of the orbitofrontal cortex (Rolls et al. 2015). The AAL2 atlas was used to delineate the anatomical regions in the brain. The AAL2 template divides the brain into 120 anatomical regions. For each region, a linear kernel was computed based on the regional pattern of activation containing all voxels within the region. The kernels were normalized (to compensate for the fact that the number of voxels varies among brain regions) and mean centered using standard operations in PRoNTo. We also trained and tested the two other classification models, support vector machine (SVM, Burges 1998) and Gaussian process classifier (GPC, Rasmussen and Williams 2006), and the two other regression models, relevance vector regression (RVR, Tipping 2001) and Gaussian process regression (GPR, Rasmussen and Williams 2006), that are currently available in PRoNTo. There were no significant differences in performance among the different classifications and regression approaches (see supplementary material). Since we were interested in identifying the contribution of each brain region for the predictive model, we present here only the results for the MKL classification and regression models.

#### Model performance

The performance of the classification models was evaluated using two metrics: balanced accuracy and area under the receiver operating characteristic (ROC) curve (AUC). In classification, it is common to compute the prediction accuracy for each class and the balanced accuracy, which is the average class accuracy. The ROC curve compares the classifier’s true positive rate (TP) and false positive rate (FP) as the decision threshold is varied. A classifier performing at chance level would, therefore, result in a 45-degree diagonal line that connects the point (0, 0) with the point (1,1), while classifiers discriminating above chance would result in an ROC curve that is ‘northwest’ of this line. The AUC is therefore a summary measure describing the performance of the classifier across all decision thresholds. A classifier achieving perfect classification would achieve an AUC of 1, while a classifier performing at chance level would achieve an AUC of 0.5.

For the performance of regression models, three metrics were used to measure the agreement between the predicted and actual threat perception scores, Pearson’s correlation coefficient (*r*), the coefficient of determination (*R*^*2*^) and the mean squared error (MSE). The correlation coefficient (*r*) describes the strength of a linear relationship between 2 variables. A small correlation is an indication of poor prediction performance. The coefficient of determination (*R*^*2*^) can be interpreted as the proportion of variance explained by the regression. The MSE is the mean of the squared differences between the predicted and true scores; it represents the mean error between the predicted and actual scores and is commonly used to evaluate the performance of predictive models.

To avoid the over-optimistic model performance that has been recently described for the leave-one-out approach (Varoquaux et al. 2017), we trained the models using two different cross-validation procedures, the “leave-one-subject-out” (LOSO) and the “k-fold-out” cross-validation (where: k = 10) methods. A nested cross-validation procedure was used to optimize the models’ hyperparamaters, with the same cross-validation scheme for the internal and external loop.

The significance of the classification and regression performance measures were determined using permutation tests, i.e., the same cross-validation procedure described above was performed 100 times with the labels permuted across the participants. The *p* value was calculated by counting how many times the absolute value of the metric with the permuted labels was equal to or bigger (smaller for MSE) than the absolute value of the metric obtained with the correct labels and dividing by 100. The results were considered significant when the obtained models performed equal to or better than the model without shuffling the labels at most 5% of the time across 100 permutation (i.e., p value < 0.05) (Schrouff et al. 2018**).**

#### Model interpretation

As previously explained, the MKL model has two sets of weights: the kernel or region weights and the voxel weights. The kernel or region weights represent the contribution of each region and the voxel weights represent the contribution of each voxel within the regions to the prediction function. Both sets of weights can be explicitly computed and plotted as brain images. Regions can then be ranked according to their contribution to the model averaged across folds. Here, we present the weight maps and a list of selected regions for the models that were statistically significant.

## Results

### Pattern classification models

The MKL classification model was able to accurately discriminate between patterns of brain activation to threat versus neutral stimuli in the *directed towards context* using both cross-validation procedures. However, the MKL classification model did not perform better than chance in discriminating between patterns of brain activation to threat versus neutral stimuli in the *directed away context* for either cross-validation procedure; all performance results are presented in Table 2. These results indicate the importance of the context directionality in the discriminability between brain responses to threat versus neural stimuli. Other classification algorithms (SVM and GPC) presented similar results. They were also able to discriminate between threat versus neutral stimuli in the directed towards context but not in the directed away context (all results are presented in supplementary materials table S-1).

**Table 2:** MKL classification model performance

Models	Cross-validation procedure	Balance accuracy	Class 1 (threat)	Class 2 (neutral)	ROC/AUC
*Directed towards threat* versus *neutral*	*LOSO*	78.95 (*p* = 0.01)	78.95 (p = 0.01)	78.95 (p = 0.01)	0.82
	*“10-fold”*	72.37 (p = 0.01)	63.16 (*p* = 0.08)	81.58 (p = 0.01)	0.78
*Directed away threat* versus *neutral*	*LOSO*	47.37 (*p* = 0.66)	47.37 (*p* = 0.67)	47.37 (*p* = 0.68)	0.49
	*“10-fold”*	60.53 (p = 0.08)	52.63 (*p* = 0.40)	68.42 (*p* = 0.03)	0.55

*p*-values were obtained by permutation test (100 permutations). *LOSO = leave-one-subject-out procedure; “10-fold” = 10-fold cross-validation procedure*

#### Regions identified by the MKL classification model

**Fig. 3:**
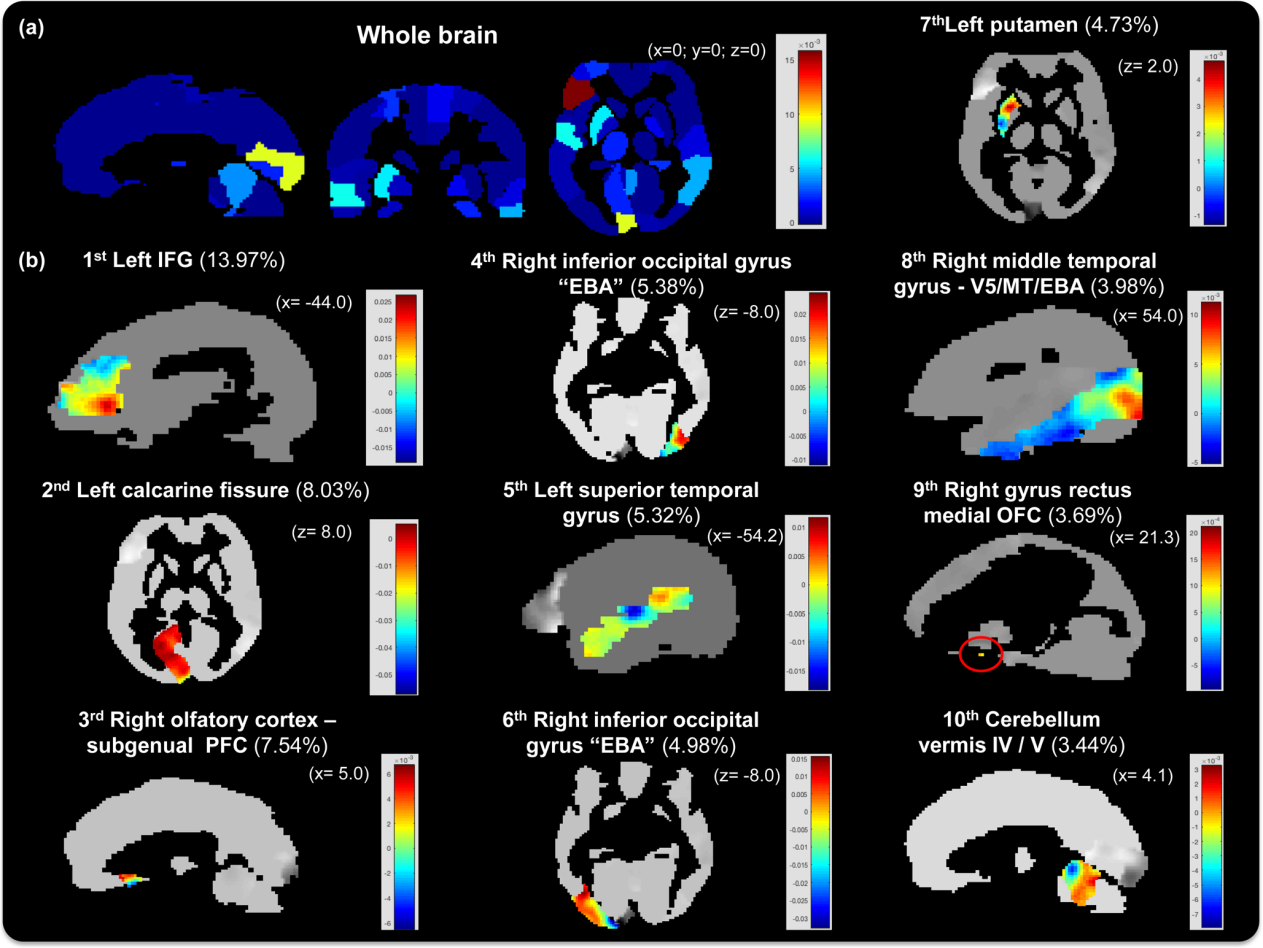
**MKL classification model based on the 10-fold cross-validation scheme:** (**a**) Whole brain map showing the kernel weights per region; the color bar represents the full range of kernel weights. (**b**) Images showing the voxels weights within the regions with highest contributions to the MKL classification model in sagittal or axial plane slices (“*x”* or “*z” MNI coordinates*, respectively). The top 10 regions ranked by the MKL classification model as relevant for discriminating between patterns of brain activity to threat versus neutral stimuli in the directed towards context are shown; the regions’ weights (in percentage) are shown in parentheses. The color bars represents the full range of voxel weights within each region. The red circle highlights small region. Acronyms: IFG – inferior frontal gyrus; EBA – extrastriate body area; PFC – prefrontal cortex; OFC – orbitofrontal cortex

When using the *10-fold cross-validation* scheme, the MKL model discriminating between patterns of brain activation to threat versus neutral stimuli in the *directed towards* context identified **60 regions** that contributed to the predictive function. When using the *LOSO cross-validation* scheme, the MKL model identified 52 regions that contributed to the predictive function. Overall the regions identified by both cross-validation schemes were similar. For the sake of brevity, we only present the regions identified using the 10-fold cross-validation scheme. The full list of regions ranked in descending order according to the kernel weights is presented in the supplementary materials table S-2. Figure 3 displays the whole-brain MKL weight map at the region level and the voxels weights for the 10 regions with the highest contributions to the predictive function.

Here, we present a summary of the kernel weights per cerebral lobe. Frontal regions had the highest contributions to the model, with a total kernel/region weights sum of 39.68%. Selected frontal regions included the left inferior frontal gyrus (triangular part), the right subgenual prefrontal cortex, the dorsolateral prefrontal cortex, and many regions of the orbitofrontal cortex. Occipital regions presented the second highest contribution to the model, with a total sum of 21.18%. Selected Occipital regions included the primary visual cortex (calcarine fissure and lingual gyrus) and more complex visual processing areas (inferior, middle and superior occipital gyrus). Some of these regions cover areas important for processing emotional body expressions (such as the extrastriate body area [EBA]). Temporal regions had the third highest kernel/regional weights, comprising 12.8%, and included three parts of the temporal gyrus (superior, middle and inferior) and Heschl’s gyrus (auditory cortex). These regions cover areas important for semantic understanding and categorical representation.

Other individual regions contributing more than 1% to the model included the left putamen (4.73%), the left parahippocampal gyrus (2.34%), the right hippocampus (2.17%), the right amygdala (2.07%), the left thalamus (1.94%), the right supplementary motor area (1.37%) and the right nucleus of the pallidum (1.25%). Regions related to emotional processing and motor response control had smaller contributions, including the left amygdala (0.68%), the right paracentral lobule (0.43%), the right middle cingulate cortex (0.21%), and the left insula (0.21%).

### Pattern regression models

**Fig. 4:**
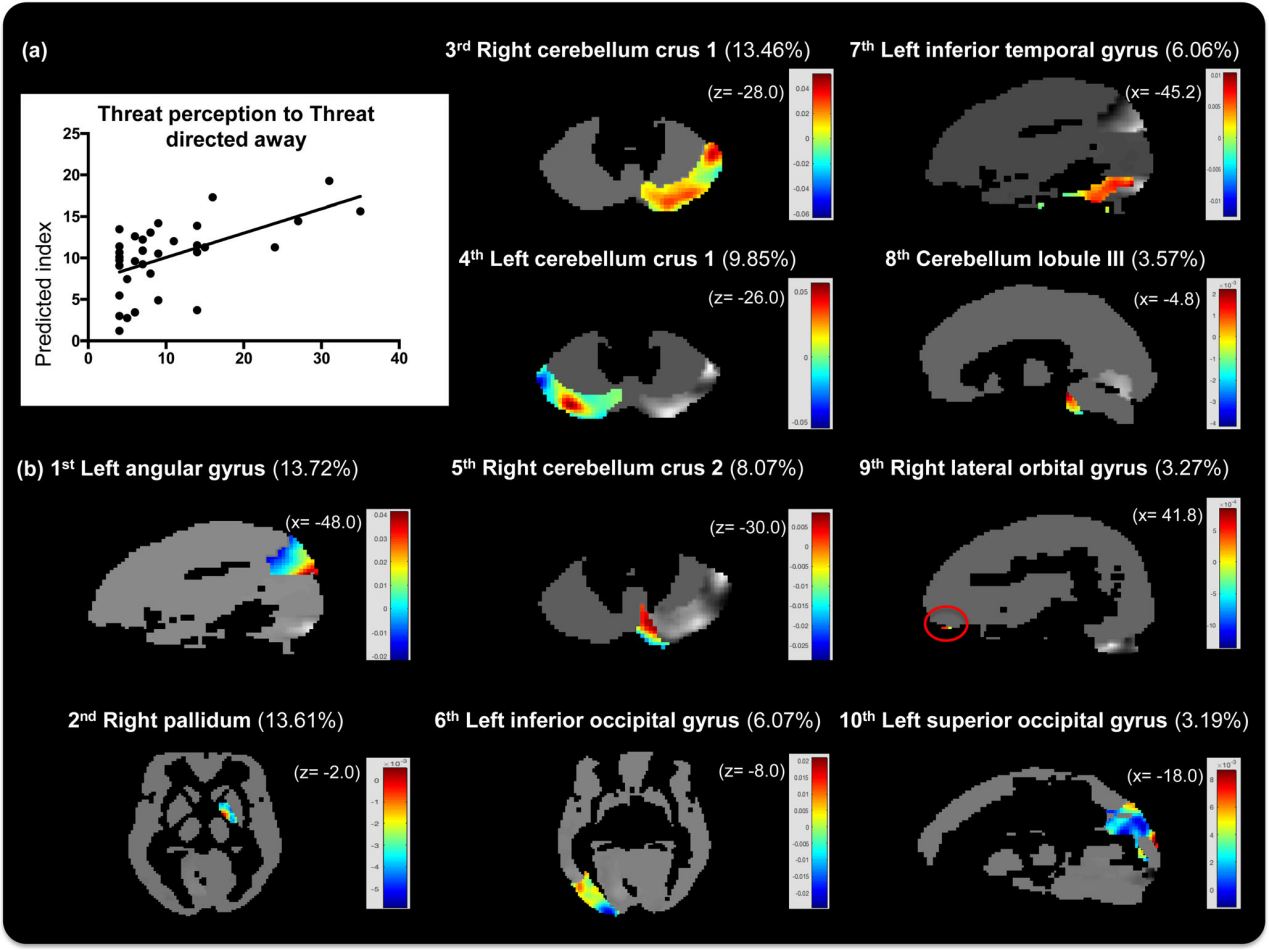
**MKL regression model based on the 10-fold cross-validation scheme:** (**a**) Scatter plot between the actual and predicted threat perception indices for the MKL regression model based on patterns of brain activation to threat stimuli in the directed away context. (**b**) Images showing the voxels weights within the regions with highest contributions to the MKL regression model in sagittal or axial plane slices (“*x”* or “*z” MNI coordinates*, respectively). The top 10 regions ranked by the MKL regression model as relevant for predicting the threat perception index are shown; the regions’ weights (in percentage) are shown in parentheses. The color bars represents the full range of voxel weights within each region. The red circle highlights small region

The results for the MKL regression models predicting threat perception index are presented in Table 3. The first model was based on patterns of brain activation to threat directed towards the observer (‘*threat directed towards regression*’), and the second model was based on patterns of brain activation to threat directed away from the observer (‘*threat directed away regression*’). The *threat directed away regression* model showed significant performance measures for both cross-validation procedures (LOSO and *10-fold cross-validation*). However, the *threat directed towards regression* model only showed significant performance for the LOSO cross-validation procedure. These results suggest that the association between patterns of brain activation to threat and the threat perception is higher when the threat is directed away from the observer. Figure 4a shows a scatter plot depicting the predicted versus actual threat perception index for the *threat directed away regression* using a 10-fold cross-validation procedure. Other regression algorithms (RVR and GPR) also showed significant performance measures for both cross-validation procedures only for the *threat directed away regression* (all results are presented in supplementary materials table S-3).

**Table 3:** MKL regression model performance

Models	Cross-validation procedure	r	R^2^	MSE
Threat directed towards regression	*LOSO*	0.48 (p = 0.03)	0.23 (*p* = 0.30)	42.94 (p = 0.03)
	*“10-fold”*	0.27 (p = 0.08)	0.07 (*p* = 0.53)	54.81 (*p* = 0.09)
Threat directed away regression	*LOSO*	0.42 (*p* = 0.02)	0.18 (*p* = 0.13)	52.24 (p = 0.02)
	*“10-fold”*	0.56 (p = 0.01)	0.31 (p = 0.01)	43.41 (*p* = 0.01)

p-values were obtained by permutation test (100 permutations). *LOSO = leave-one-subject-out procedure; “10-fold” = 10-fold cross-validation procedure*

#### Regions identified by the MKL regression model

When using the *10-fold cross-validation* scheme, the *threat directed away regression* MKL model identified **43 regions** that contributed to the predictive function. When using the *LOSO cross-validation* scheme, the MKL model identified 38 regions that contributed to the predictive function. Overall the regions identified by both cross-validation schemes were similar. For the sake of brevity, we only present the regions identified using the 10-fold cross-validation scheme. The full list of regions ranked in descending order according to the kernel weights is presented in the supplementary materials in table S-4. Figure 4b shows the whole-brain MKL weight map at the region level and the voxels weights for the 10 regions with the highest contributions to the predictive function.

Here, we presented a summary of the kernel weights per cerebral lobes. Cerebellum regions presented a kernel weight sum of 23.97%. Occipital regions contributed 14.56% to the model. Selected occipital regions included complex visual processing areas (such as the superior and inferior occipital gyri) and early processing visual areas (such as the lingual gyrus and the right calcarine fissure). Frontal regions presented a total kernel weight sum of 10.01%. Selected frontal regions included the right lateral orbital gyrus, the right subgenual prefrontal cortex, and the right dorsolateral superior frontal gyrus. Temporal regions presented a total kernel weight sum of 7.64%. Selected temporal regions included the left inferior temporal gyrus, the left middle temporal gyrus, and the right Heschl’s gyrus (auditory cortex). Subcortical gray nuclei regions had a total kernel weight sum of 1.8%. Other regions had kernel weight contributions below 1%.

## Discussion

The main goal of the present study was to investigate whether the discriminability between patterns of brain activation to threat versus neutral stimuli measured by the MVPA performance (i.e., accuracy) could be influenced by subtle differences in the emotional context due to the directionality of the stimuli. Our results demonstrate that changes in the directionality of the stimuli affect the performance of MVPA algorithms. In the classification model, a significant accuracy for discriminating brain activation patterns to threat versus neutral stimuli was found only in the directed towards context. The classification model was not able to discriminate between patterns of brain activation to threat versus neutral stimuli in the directed away context. These results demonstrate that the emotional context plays an important role in the discriminability of brain activation patterns to emotional stimuli. These findings agree with the idea that the direction of the threat stimulus is a critical factor to modulate brain responses to an emotional stimulus. Additionally, we investigated whether the subjective measure of threat perception could be decoded from patterns of whole-brain activity to threatening stimuli in different contexts using pattern regression analysis. Interestingly, our results showed that it was possible to decode the individuals’ subjective threat perceptions from patterns of brain activation to threat, with a better performance in the directed away threat context than in the directed towards context. In summary, these results suggest that in the directed towards context, the patterns of brain activation to threat and neutral stimuli are more consistent across individuals, which improves the performance of classification models in discriminating between the patterns of brain activation to these stimuli (threat versus neutral). Conversely, the greater variability across individuals in the direct away context, potentially associated with subjective threat perception, facilitates the regression model learning.

The ability to discriminate between patterns of brain activation to threat versus neutral stimuli obtained in the directed towards contexts is in line with previous studies in the literature. Most studies have shown discrimination between brain activation to emotional versus neutral stimuli using various emotions expressed by faces (Harry et al. 2013; Pessoa and Padmala 2007; Wegrzyn et al. 2015), videos clips, audio sounds, words (Ethofer et al. 2009; Markey et al. 2013; Oosterwijk et al. 2017; Skerry and Saxe 2015), and emotional pictures from IAPS with a wide range of valence and arousal ratings (Baucom et al. 2012; Yuen et al. 2012).

Threat stimuli directed towards the observer has been shown to be an important factor for activating more intense emotional responses. A number of studies in the literature have shown that the direction of the threat can modulate psychophysiological responses and behavioral measures (Bastos et al. 2016; Carlson et al. 2009; Dimberg and Ohman 1983; Fernandes et al. 2013; Flykt et al. 2007; Hugdahl and Johnsen 1989). For instance, previous studies have shown increased activity in skin conduce responses to directed angry faces during conditioning (Dimberg and Ohman 1983), reduced extinction during fear conditioning to directed towards stimuli of snakes and guns (Flykt et al. 2007; Hugdahl and Johnsen 1989), and improved spatial attention to threat stimuli directed towards the viewer (such as a pointed gun and striking snake) on a dot-probe test (Carlson et al. 2009). Additionally, Hortensius and colleagues (Hortensius et al. 2016) showed that the direction of threat differently influences explicit recognition. Recognition accuracy was higher for anger directed toward the observer than for anger directed away from the observer, while the opposite pattern was found for fearful expressions. In the same vein, our group has shown that threat stimuli directed towards the observer can activate more intense defensive cascade responses (Bastos et al. 2016; Fernandes et al. 2013). More specifically, Fernandes et al. (2013) found that threat stimuli directed towards the observer induced a decreased reaction time (RT) while participants performed a bar orientation discrimination task. This accelerated RT during the directed towards threat stimuli scenario was attributed to increased motor preparation resulting from an intense defensive response. Thus, one possible explanation for the better performance of the MKL model in discriminating between patterns of brain responses to threat versus neutral stimuli in the directed towards context could be attributed to an increase in threat perception, which could lead to more consistently increased brain activation across subjects and less susceptibility of the model to individual variability. In fact, the MKL regression model was not able to consistently decode the threat perception index of individuals from patterns of brain activation to threat during the directed towards context, suggesting that the patterns of brain activation during this context were not strongly associated with the individuals’ subjective threat perceptions. This effect is likely because the threat stimuli in the directed towards context are more threatening and may evoke more homogeneous emotional reactions across subjects.

The MKL classification algorithm was not able to discriminate between patterns of brain activation to threat versus neutral stimuli in the directed away context. A previous study reported by our group showed that threat stimuli directed away from the viewer were considered less intense, farther away, and more escapable than threat stimuli directed towards the viewer (Fernandes et al. 2013). These stimuli were also classified as more ambiguous than the directed towards stimuli (Bastos et al. 2016). One possible explanation for this finding is that the directed away stimuli produced patterns of brain activation that were more heterogeneous across subjects and more susceptible to individual variability. In agreement with our hypothesis, previous work from our group showed that MKL regression analyses were able to decode negative affect (NA) from patterns of brain activation to threat in the directed away context but not in the directed towards context (Fernandes et al. 2017). These results indicate that the patterns of brain activation to threatening stimuli during the directed away context are more influenced by individual variability.

The MKL classification model discriminating between patterns of brain activation to threat versus neutral stimuli in the *directed towards* context identified 60 brain regions from the AAL2 atlas (Rolls et al. 2015) that were relevant for the classification. The top 10 regions corresponded to a sum of 61.1% of the total kernel weights. The brain regions with the highest contributions to the MKL classification model included the prefrontal cortex (left inferior frontal gyrus, right subgenual prefrontal cortex, and medial orbitofrontal cortex), the left putamen, the cerebellum, occipital and temporal regions. Prefrontal regions contributed to 39.68% of the total kernel weights. These results are consistent with the literature, as prefrontal regions play more important roles in decoding emotional information by evaluating the emotional content of stimuli and selecting the best defensive strategy according to each context (Mobbs et al. 2007, 2009, 2010). Occipitotemporal regions contributed to 23.0% of the kernel weights and included the EBA, the superior temporal sulcus (STS), and the middle temporal/V5 complex (MT/V5, or motion area) in the middle temporal gyrus. The EBA has been shown to exhibit strong and selective responses to images of human bodies and body parts (Downing et al. 2001). Many studies have also reported EBA activity to static and dynamic emotional stimuli (Grèzes et al. 2007; Kret et al. 2011; Peelen et al. 2007). The STS region has been shown to be activated in face perception processing (Haxby et al. 2000). Furthermore, connections from the STS to structures including the amygdala (Kravitz et al. 2013) suggest that this network mediates the evaluation of visual stimuli in emotional responses. Our findings provide further evidence that the functions of the EBA and the STS go beyond the mere perception of body shape, reinforcing the idea that these areas are sensitive to threating body/facial expressions and the violence expressed by a conspecific.

The MKL regression model used to decode the individuals’ threat perception from patterns of brain activation to threat in the *directed away* context identified 43 regions distributed across the brain as relevant for the predictive function. The top 10 regions corresponded to a sum of 80.9% of the total kernel weights. The region with the highest contribution to the MKL regression model was the left angular gyrus in the parietal cortex (13.7% kernel weight). The role of this region seems to be related to processing concepts (e.g., when a motor response is requested after a stimulus) rather than perception (Seghier 2012). In fact, the left angular gyrus is considered a cross-modal hub where converging multisensory information is combined and integrated to solve problems (Seghier 2012). Engelen and colleagues (Engelen et al. 2015) found that the inferior parietal lobule plays a critical role in emotional body processing. Threat perception may have potentially modulated the activity of this region: Individuals who evaluated the threat stimuli *directed away* as more threatening may have had a different activation pattern than individuals who evaluated the threat stimuli directed away as less threatening. Taken together, these findings support the importance of the angular gyrus in the parietal lobule in decoding subjective perceptions of threat from patterns of brain activation to threatening stimuli in the *directed away* context.

This study has some limitations. First, the results of our classification models were obtained with a sample of 38 participants, and the results of our regression models were obtained with a sample of 34 participants; therefore, the conclusions cannot be extrapolated as representative of large populations. Even though we tested different cross-validation procedures, the models should ideally be trained and tested with truly independent samples. Further studies with larger sample sizes are needed to assess the generalizability of these results by training and testing the models with completely independent samples. Another limitation of this study is the fact in the MKL classification and regression models the sparsity on the number of selected kernels (or regions in the present work) was imposed by an L1-norm regularization constraint, which might not select regions with correlated information. Future studies should explore other MKL approaches, including a combination of L1- and L2-norm regularization constraints to address this limitation. Finally, threat stimuli *directed towards* the individuals were considered more arousing than threat stimuli *directed away*. Threat stimuli *directed toward* the individuals were expected to produce more intense activation of the defensive cascade than threat stimuli *directed away* (Fernandes et al. 2013), and an increase in arousal is inevitable. Furthermore, Kragel and LaBar (2015) demonstrated that differences in categorical emotional states, as represented by neural activation patterns, are more separable by multivariate classification analyses than differences in terms of valence or arousal. Therefore, although we cannot rule out the contribution of arousal, it is unlikely that the results of the classification models were completely explained by differences in arousal. Additional studies should be performed to investigate this issue.

In conclusion, we observed that the directionality of the stimulus plays an important role in the discriminability between patterns of brain activation to threat versus neutral stimuli and in decoding individuals’ subjective threat perceptions from patterns of brain activation to threat. Furthermore, we found a double dissociation effect related to the emotional context: The *directed towards* context enabled discriminating between patterns of brain activation to threat versus neutral stimuli but not decoding the individuals’ subjective threat perceptions from patterns of brain activation to threat. Conversely, the *directed away* context facilitated better performance in decoding the individuals’ subjective threat perceptions but not in discriminating between patterns of brain activation to threat versus neutral stimuli. One possible explanation for this dissociation is that, in the *directed towards* context, the threat perception produced a more intense defensive reaction, inducing more homogeneous patterns of brain activation across individuals and, thus, facilitating the classification task. Conversely, in the *directed away* context, the threat perception produced a less intense and more variable defensive reaction among individuals, capturing individual differences and facilitating the regression model learning. Finally, as a concluding remark, the findings of the present study corroborate the idea that the direction of the threat stimulus is a critical factor that modulates the brain response to an emotional stimulus. Furthermore, we provide evidence that prefrontal regions, including the EBA and the STS, are relevant brain regions to decode the direction of the threat.

## Electronic supplementary material

ESM 1 (DOCX 67 kb)
