# Supplementary material for: How do you perceive threat? It’s all in your pattern of brain activity
Source: Brain Imaging Behav. 2019 Aug 24;14(6):2251–66. doi: 10.1007/s11682-019-00177-6 (PMC7648008; doi:10.1007/s11682-019-00177-6)
Supplement: Supplementary file 1 — (DOCX 67 kb) [file 11682_2019_177_MOESM1_ESM.docx]

**Supplementary materials**

**Table S-1 – Machines tests for classification models**

| **Classification Models** | **Machines** | **Cross-validation schemes** | **Balance Accuracy** | **Class 1** | **Class 2** | **ROC/AUC** |
| --- | --- | --- | --- | --- | --- | --- |
| **Directed towards threat vs. neutral** | SVM* | LOSO | 72.37 (p=0.004) | 71.05 (p=0.01) | 73.68 (p=0.004) | 0.81 |
|  |  | "10-folds" | 76.32 (p=0.001) | 73.68 (p=0.003) | 78.95 (p=0.001) | 0.81 |
|  | GPC | LOSO | 71.05 (p=0.01) | 71.05 (p=0.01) | 71.05 (p=0.01) | 0.83 |
|  |  | "10-folds" | 75 (p=0.01) | 73.68 (p=0.01) | 76.32 (p=0.01) | 0.82 |
| **Directed away threat vs. neutral** | SVM* | LOSO | 60.53 (p=0.103) | 57.89 (p=0.201) | 63.16 (p=0.073) | 0.66 |
|  |  | "10-folds" | 57.89 (p=0.179) | 57.89 (p=0.215) | 57.89 (p=0.23) | 0.66 |
|  | GPC | LOSO | 60.53 (p=0.11) | 63.16 (p=0.10) | 57.89 (p=0.17) | 0.63 |
|  |  | "10-folds" | 56.58 (p=0.31) | 57.89 (p=0.29) | 55.26 (p=0.42) | 0.65 |

Note: p-value was obtained by permutation test (100 permutations and *1000 permutation). *LOSO= leave-one-subject-out; “10-folds”= 10 folds cross-validation scheme.*

**Table S-2 – MKL Classification model:** Brain regions ranked according to their contribution to the prediction function for discriminating between threat versus neutral stimuli in the directed towards context.

| **Rank** | **REGIONS** | **ROI**  **kernel weight (%)** | **ROI size**  **(voxel)** |
| --- | --- | --- | --- |
| **1** | Inferior frontal gyrus L, triangular part | 13.97 | 2190 |
| **2** | Calcarine fissure L (early visual cortex) | 8.03 | 1929 |
| **3** | Olfactory cortex R (subgenual PFC) | 7.54 | 89 |
| **4** | Inferior occipital gyrus L (EBA) | 5.38 | 653 |
| **5** | Superior temporal gyrus L | 5.32 | 2182 |
| **6** | Inferior occipital gyrus R (EBA) | 4.98 | 789 |
| **7** | Putamen L | 4.73 | 1008 |
| **8** | Middle temporal gyrus R (V5/MT/EBA) | 3.98 | 3133 |
| **9** | Gyrus rectus R (medial orbitofrontal cortex) | 3.69 | 6 |
| **10** | Cerebellum vermis IV/V | 3.44 | 665 |
| **11** | Cerebellum vermis X | 2.52 | 34 |
| **12** | Parahippocampal gyrus L | 2.34 | 403 |
| **13** | Posterior orbital gyrus L (orbitofrontal cortex) | 2.18 | 180 |
| **14** | Hippocampus R | 2.17 | 905 |
| **15** | Superior frontal gyrus L, dorsolateral | 2.14 | 3265 |
| **16** | Amygdala R | 2.07 | 167 |
| **17** | Lingual gyrus (early visual cortex) | 2.06 | 1957 |
| **18** | Anterior orbital gyrus R (orbitofrontal cortex) | 2.06 | 60 |
| **19** | Thalamus L | 1.94 | 1100 |
| **20** | Lateral orbital gyrus L (orbitofrontal cortex) | 1.84 | 27 |
| **21** | Inferior Frontal Gyrus L, pars orbitalis | 1.65 | 686 |
| **22** | Supplementary motor area | 1.37 | 2003 |
| **23** | Pallidum R | 1.25 | 280 |
| **24** | Temporal pole, middle temporal gyrus R | 1.17 | 52 |
| **25** | Middle frontal gyrus L (dorsolateral prefrontal cortex) | 1.13 | 3268 |
| **26** | Superior temporal gyrus R | 1.00 | 2468 |
| **27** | Posterior orbital gyrus R (orbitofrontal cortex) | 0.88 | 174 |
| **28** | Amygdala L | 0.68 | 167 |
| **29** | Pallidum L | 0.65 | 293 |
| **30** | Olfactory cortex L (subgenulal PFC) | 0.62 | 90 |
| **31** | Cerebellum vermis I/II | 0.61 | 53 |
| **32** | Cerebellum vermis IX | 0.60 | 20 |
| **33** | Inferior Frontal Gyrus R, pars orbitalis | 0.59 | 568 |
| **34** | Rolandic opperculum L | 0.54 | 947 |
| **35** | Middle temporal gyrus L (V5/MT/EBA) | 0.54 | 3740 |
| **36** | Calcarine fissure R (visual cortex) | 0.50 | 1773 |
| **37** | Superior frontal gyrus R, dorsolateral | 0.45 | 3713 |
| **38** | Paracentral lobule L (primary motor and premotor cortex) | 0.43 | 709 |
| **39** | Heschl's gyrus (auditory cortex) | 0.36 | 224 |
| **40** | Cerebellum hemispheric lobule IV/V | 0.35 | 975 |
| **41** | Inferior temporal gyrus L | 0.24 | 639 |
| **42** | Cerebellum hemispheric lobule VIII L | 0.24 | 10 |
| **43** | Middle occipital gyrus R | 0.22 | 1551 |
| **44** | Middle cingulate cortex R | 0.21 | 2203 |
| **45** | Insula cortex L | 0.21 | 1832 |
| **46** | Parahippocampal gyrus R | 0.14 | 499 |
| **47** | Cerebellum crus | 0.14 | 975 |
| **48** | Temporal pole, supperior temporal gyrus L | 0.14 | 467 |
| **49** | Anterior orbital gyrus L (orbitofrontal cortex) | 0.14 | 47 |
| **50** | Lateral orbital gyrus R (orbitofrontal cortex) | 0.13 | 10 |
| **51** | Cerebellum hemispheric lobule VIII R | 0.12 | 9 |
| **52** | Cerebellum vermis III | 0.09 | 228 |
| **53** | Superior frontal gyrus, medial orbital | 0.07 | 399 |
| **54** | Superior frontal gyrus, medial | 0.05 | 1560 |
| **55** | Temporal pole R, superior temporal gyrus | 0.04 | 416 |
| **56** | Anterior cingulate cortex R | 0.03 | 1313 |
| **57** | Medial orbital gyrus R (orbitofrontal cortex) | 0.02 | 1 |
| **58** | Cerebellum vermis VII | 0.006 | 176 |
| **59** | Posterior cingulate cortex L | 0.006 | 463 |
| **60** | Paracentral lobule R (primary motor and premotor cortex) | 0.002 | 443 |

Note: L=left and R= right

**Table S-3 – Machines tests for regression models**

| **Regression Models** | **Machines** | **Cross-validation schemes** | **r** | ***R^2^*** | **MSE** |
| --- | --- | --- | --- | --- | --- |
| **Threat directed toward regression** | RVR | LOSO | -0.01  (p=0.36) | 0.00 (p=0.94) | 64.33 (p=0.30) |
|  |  | "10-folds" | -0.11 (p=0.53) | 0.01 (p=0.65) | 73.82 (p=0.56) |
|  | GPR | LOSO | -0.01 (p=0.46) | 0.00 (p=0.99) | 68.62 (p=0.47) |
|  |  | "10-folds" | -0.11 (p=0.55) | 0.01 (p=0.65) | 73.82 (p=0.55) |
| **Threat directed away regression** | RVR | LOSO | 0.27 (p=0.03) | 0.07 (p=0.14) | 60.11 (p=0.03) |
|  |  | "10-folds" | 0.32 (p=0.05) | 0.10 (p=0.12) | 56.70 (p=0.05) |
|  | GPR | LOSO | 0.27 (p=0.07) | 0.07 (p=0.28) | 60.04 (p=0.06) |
|  |  | "10-folds" | 0.28 (p=0.07) | 0.08 (p=0.64) | 58.85 (p=0.07) |

Note: p-value was obtained by permutation test (100 permutations). *LOSO= leave-one-subject-out; “10-folds”= 10 folds cross-validation scheme.*

**Table S-4 – MKL Regression model:** Brain regions ranked according to their contribution to the predicted function for predicting the threat perception index from patterns of brain activation to threat stimuli in the directed away context.

| **Rank** | **REGIONS** | **ROI kernel weight (%)** | **ROI size**  **(voxel)** |
| --- | --- | --- | --- |
| **1** | Angular gyrus L | 13.72 | 1106 |
| **2** | Pallidum R | 13.61 | 280 |
| **3** | Cerebellum crus 1 R | 13.46 | 982 |
| **4** | Cerebellum crus 1 L | 9.85 | 1084 |
| **5** | Cerebellum crus 2 R | 8.07 | 79 |
| **6** | Inferior occipital gyrus L (EBA) | 6.07 | 791 |
| **7** | Inferior temporal gyrus L | 6.06 | 675 |
| **8** | Cerebellum hemispheric lobule III | 3.57 | 136 |
| **9** | Lateral orbital gyrus (orbitofrontal cortex) | 3.27 | 10 |
| **10** | Supperior occipital gyrus | 3.19 | 987 |
| **11** | Olfactory cortex R (subgenual PFC) | 2.88 | 90 |
| **12** | Lingual gyrus (Visual Cortex) | 2.10 | 2140 |
| **13** | Lingual gyrus (Visual Cortex) | 1.98 | 1965 |
| **14** | Putamen L | 1.17 | 1008 |
| **15** | Superior frontal gyrus R, dorlateral | 1.08 | 3713 |
| **16** | Middle temporal gyrus (V5/MT/EBA) | 0.89 | 3740 |
| **17** | Superior frontal gyrus, medial | 0.83 | 1560 |
| **18** | Cerebellum hemispheric lobule IV/V | 0.74 | 975 |
| **19** | Fissure calcarine | 0.70 | 1773 |
| **20** | Heschl's gyrus (auditory cortex) | 0.69 | 241 |
| **21** | Cerebellum crus 2 L | 0.64 | 141 |
| **22** | Cerebellum vermis IX | 0.62 | 20 |
| **23** | Superior frontal gyrus, medial | 0.46 | 2290 |
| **24** | IFG pars orbitalis | 0.43 | 686 |
| **25** | Thalamus | 0.42 | 1100 |
| **26** | Inferior frontal gyrus, triangular part | 0.39 | 2190 |
| **27** | Paracentral lobule (primary motor and premotor cortex) | 0.34 | 719 |
| **28** | Paracentral lobule (primary motor and premotor cortex) | 0.32 | 454 |
| **29** | Inferior occipital gyrus R (EBA) | 0.31 | 682 |
| **30** | Anterior orbital gyrus (orbitofrontal cortex) | 0.30 | 47 |
| **31** | Cerebellum hemispheric lobule VI | 0.23 | 1394 |
| **32** | Angular gyrus L | 0.22 | 1381 |
| **33** | Gyrus rectus (medial obritofrontal cortex) | 0.21 | 6 |
| **34** | Supperior occipital gyrus | 0.21 | 1041 |
| **35** | Amygdala R | 0.20 | 172 |
| **36** | Middle cingulate cortex R | 0.18 | 2203 |
| **37** | Gyrus rectus (medial obritofrontal cortex) | 0.14 | 14 |
| **38** | Cerebellum vermis VI | 0.12 | 371 |
| **39** | Cerebellum vermis X | 0.094 | 34 |
| **40** | Posterior orbital gyrus | 0.082 | 174 |
| **41** | Postcentral gyrus | 0.080 | 3028 |
| **42** | Cerebellum hemispheric lobule VI | 0.029 | 1473 |
| **43** | Parahippocampal gyrus | 0.004 | 503 |

Note: L=left and R= right
